# Sagittal collimating diaboloid: a new grazing-incidence mirror surface for higher-throughput resonant inelastic X-ray scattering spectrometers

**DOI:** 10.1107/S1600577526005564

**Published:** 2026-06-29

**Authors:** Joseph Dvorak, Lei Huang, Mourad Idir

**Affiliations:** a National Synchrotron Light Source II Brookhaven National Laboratory PO Box 5000 Upton NY11973 USA; Tohoku University, Japan

**Keywords:** synchrotron optics, diaboloids, toroids, X-ray spectrometers

## Abstract

A major challenge in soft X-ray spectroscopy is the efficient collection of the emitted X-rays by grazing-incidence mirrors. In this energy range, grazing-incidence mirrors are widely used as optics for the collection of light. The small angle of incidence necessarily limits the collection solid angle of soft X-ray spectrometers. We present a new mirror surface, a sagittal collimating diaboloid, that can both collect and focus light from a point source in an aberration-free manner. The usefulness of this optic in increasing throughput is demonstrated with a realistic example design of a moderate-sized (3 m) medium-resolution resonant inelastic X-ray scattering spectrometer.

## Abstract 2

We present the analytic equations for a new grazing-incidence mirror surface, a sagittal collimating diaboloid, and illustrate its advantages for higher-throughput soft X-ray spectrometer design.

## 1. Introduction

Soft X-ray fluorescence and resonant inelastic X-ray scattering (RIXS) are inherently low cross section events, such that they are invariably carried out at high-brilliance sources such as synchrotrons and free-electron lasers. In the case of fluorescence, only a few percent of excited core-holes decay via emission of a photon, the rest decaying through non-radiative processes involving electron emission. For instance, both the *K* shell fluorescence efficiency of the important light elements (Li to Ne) is less than 2% and the *L* shell fluorescence efficiency of the 3*d* transition metals is less than 1% (Kortright, 2009 ▸).

While quantitative measurements of absolute RIXS cross sections are hard to come by, estimates of existing spectrometer efficiencies can be made. Take, for instance, the RIXS spectrometer on the SIX beamline at National Synchrotron Light Source II (NSLS II; Dvorak *et al.*, 2016 ▸). It is designed for high efficiency, with two parabolic mirrors close to the sample for high collection efficiency. Each mirror has a horizontal acceptance of 30 mrad. The mirrors are oriented face to face, which allows two beams to be collected from the sample simultaneously, thus doubling the throughput. The horizontal collection angle of the spectrometer is then 60 mrad. Even so, when operating in ultra-high resolution mode, only about one inelastically scattering event in 10^11^ photons incident on the sample is collected by the spectrometer on a specific excitation peak (Bisogni, 2025 ▸).

Another consideration motivating improved spectrometer design is the problem of sample damage. Focus sizes of the order of 1–10 µm are commonly obtained at current spectroscopy endstations (Ghiringhelli *et al.*, 2006 ▸; Harada *et al.*, 2012 ▸; Könnecke *et al.*, 2013 ▸; Yin *et al.*, 2015 ▸; Lieutenant *et al.*, 2016 ▸; Dvorak *et al.*, 2016 ▸; Qiao *et al.*, 2017 ▸; Brookes *et al.*, 2018 ▸; Singh *et al.*, 2021 ▸; Zhou *et al.*, 2022 ▸; Weinhardt *et al.*, 2024 ▸) with the trend moving towards submicrometre focus sizes. As the flux density scales as the inverse square of the focus size, the problem of sample damage becomes increasingly prevalent, especially for molecular systems such as those found in chemistry- and biology-relevant samples (Weinhardt *et al.*, 2013 ▸; Schmitt *et al.*, 2014 ▸). This argues strongly towards increasing spectrometer efficiency, rather than the brute force approach of increasing the beam flux on the sample.

**Figure 1:**
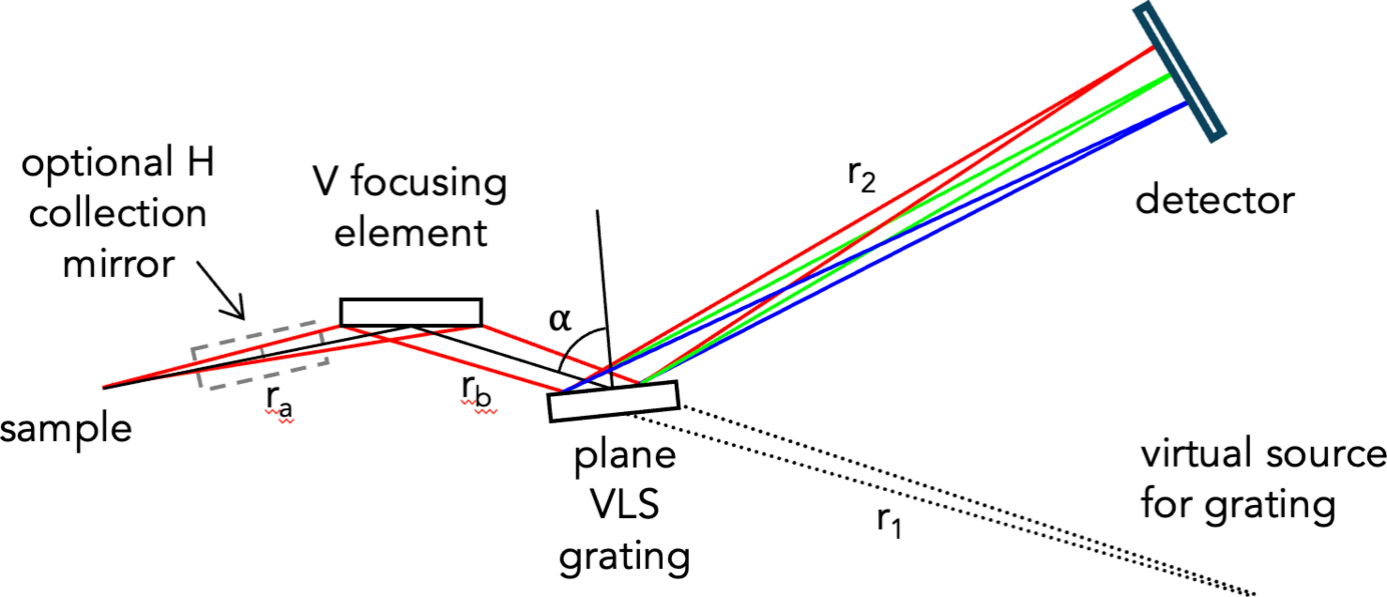
Side view schematic diagram of a Hettrick–Underwood spectrometer.

In the light of these considerations, anything that can be done to improve the throughput of soft X-ray spectrometers is welcome. Reducing the number of optics and increasing the acceptance solid angle are two strategies to attempt. To that end, we introduce here a new mirror surface, a sagittal collimating diaboloid. We take as our starting point the Hettrick–Underwood spectrometer design (Hettrick & Underwood, 1986 ▸; Hettrick, 1988 ▸; Hettrick *et al.*, 1988 ▸), a schematic diagram of which is shown in Fig. 1 ▸. In the Hettrick–Underwood design, a mirror upstream of the grating collects light in the vertical direction^1^ and vertically focuses from the source to some point *r*_1_ beyond the grating. The variable line space (VLS) grating then disperses the light at the detector and reduces aberrations. The usual choice is to make *r*_1_ = −*r*_2_, the negative value indicating that the source is virtual from the point of view of the grating. While the Hettrick–Underwood design is not the only possible soft X-ray spectrometer design, it has several well known features that make it a good choice, as it has an upright focal plane and the distance from the grating to the focus *r*_2_ varies little with wavelength. The Hettrick–Underwood design has been implemented in many soft X-ray spectrometers (Hague *et al.*, 2005 ▸; Fuchs *et al.*, 2009 ▸; Chiuzbăian *et al.*, 2014 ▸; Dvorak *et al.*, 2016 ▸; Qiao *et al.*, 2017 ▸).

Now comes the issue of throughput. For medium- to high-resolution spectrometers [resolving power (RP) ≳1000], the acceptance in the vertical direction is limited to the order of 10 mrad in positive diffraction order (outside order) due to the requirement that the grating be able to scan over a wide energy range. For vertical acceptances larger than this, the resolution degrades rapidly as the acceptance is increased, due to incomplete cancelation of the aberrations by the VLS grating. It is possible to increase the vertical acceptance at the expense of energy resolution by operating the grating in negative diffraction order (inside order), and this is commonly done for lower resolution spectrometers.

That leaves the horizontal direction available to increase the acceptance. For shorter spectrometers, often no optics are included to increase the acceptance in the horizontal direction; the horizontal acceptance is then determined by the detector width. This simple choice is used for a variety of spectrometers (Ghiringhelli *et al.*, 2006 ▸; Fuchs *et al.*, 2009 ▸; Harada *et al.*, 2012 ▸; Yin *et al.*, 2015 ▸; Lieutenant *et al.*, 2016 ▸; Qiao *et al.*, 2017 ▸; Weinhardt *et al.*, 2024 ▸). However, due to the low efficiency of the RIXS process, this approach becomes less attractive the larger the spectrometer, as the acceptance in the non-dispersion direction falls as the inverse of the distance from the source for a fixed detector width.

The horizontal collection angle can be increased by adding a horizontal collection mirror, as shown in Fig. 1 ▸. To minimize the resolution degradation due to figure error, the mirror is typically oriented to deflect the beam horizontally and it collects the beam along the tangential direction. This requires a relatively long mirror for large acceptance. The horizontal collection mirror is implemented in many spectrometers (Hague *et al.*, 2005 ▸; Könnecke *et al.*, 2013 ▸; Chiuzbăian *et al.*, 2014 ▸; Dvorak *et al.*, 2016 ▸; Brookes *et al.*, 2018 ▸; Singh *et al.*, 2021 ▸; Zhou *et al.*, 2022 ▸; MAX IV, 2025 ▸).

To illustrate the effectiveness of this approach, we look to the Centurion ultrahigh resolution RIXS spectrometer on the SIX beamline at NSLS II (Dvorak *et al.*, 2016 ▸). This spectrometer has a horizontal deflection parabolic mirror for beam collection close to the source. The mirror is 250 mm long and is located 275 mm from the source at a grazing-incidence angle of 1.5° for a horizontal acceptance of 27.5 mrad. This mirror collimates the light into an 8 mm wide beam that fits easily onto a standard CCD detector. If left to propagate uncollimated down the 15 m length of the spectrometer, this beam would expand to about 400 mm wide, requiring 16 tiled detectors for the same acceptance, assuming a common 25 mm wide detector chip.

The horizontal collection mirror has limitations on its effectiveness, however. In certain situations, it is possible to place it close to the sample inside the sample chamber (Dvorak *et al.*, 2016 ▸; Zhou *et al.*, 2022 ▸), in which case the length can be kept relatively short. However, there are many instances where the space near the sample is too crowded to fit additional optics, or the sample is exposed to reactive gases which preclude having optics inside the chamber. In that case, the mirror must be placed outside the sample chamber, and the mirror length must increase proportionally to the distance from the sample to have the same acceptance. For long spectrometers this space may be available, but for short spectrometers this will push the vertical focusing mirror (VFM) and grating further downstream, decreasing the vertical acceptance.

All of the above brings us to the idea of putting sagittal curvature on the vertical focusing mirror. Such an optic could efficiently collect light in the horizontal direction while simultaneously focusing the light in the vertical direction, eliminating the need for a separate horizontal collection mirror. The first figure that comes to mind is a toroid. However, toroids are known to perform well only in a 1:1 geometry, and in one special configuration in which a toroid is used to focus an incoming cylindrical wave with a 2:1 ratio in the sagittal direction (MacDowell *et al.*, 2004 ▸). This is because two of the lower-order aberration terms in the path function expression, *F*_400_ and *F*_040_, are zeroed when the source and image distances are equal (Howells, 2009 ▸). While the VLS law on the grating can be used to correct for tangential aberrations introduced by a toroid in a non 1:1 geometry, there is no such correction in the sagittal direction. Intuition tells us that the aberrations introduced by the sagittal curvature of the toroid will rapidly degrade the focusing, and hence the energy resolution, at the tens of milliradian acceptances we are aiming for. What is needed is a new mirror surface figure: a sagittal collimating diaboloid.

**Figure 2:**
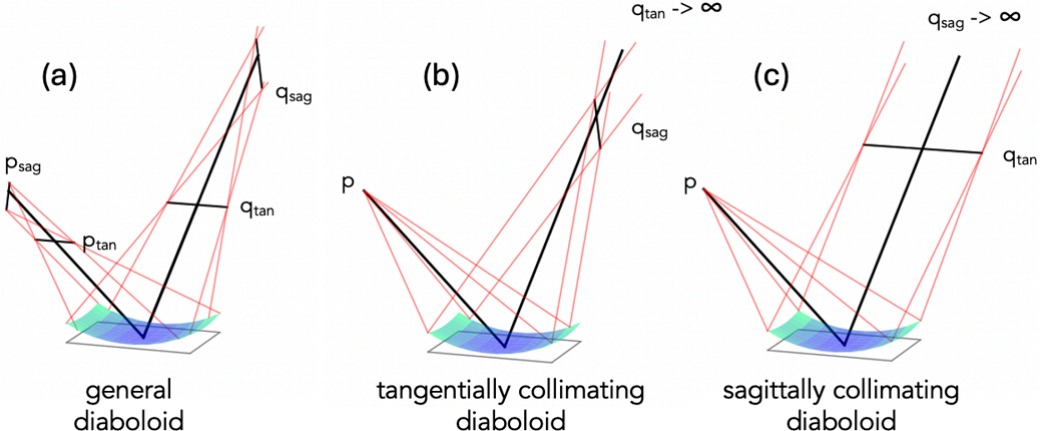
Schematic diagrams of several diaboloids. A general diaboloid has separate source and image distances for the tangential and sagittal line foci. A tangentially collimating diaboloid is formed by letting the tangential and sagittal source distances coincide, in which case the source coalesces into a point, and by letting the tangential image distance go to infinity. A sagittal focusing diaboloid is formed in a similar manner except the sagittal image distance is set to infinity. Note that the tangential direction is parallel to the plane of incidence defined by the main incident and reflected rays (black lines) in the diagrams; the sagittal direction is perpendicular to the plane of incidence.

What is a diaboloid? It can be thought of as an aberration-corrected toroid. A formal definition has not yet been put forth. We propose the following: A generalized diaboloid is an optic that focuses, without aberrations, separate tangential and sagittal line sources into separate tangential and sagittal line images. When the tangential and sagittal source/image distances coincide, they coalesce into a point. This is not the first reference to a diaboloid or similar optic. Free-form mirrors have been discussed previously to deal with the deep and curved source presented by a synchrotron bending magnet (López-Delgado & Szwarc, 1976 ▸). In addition, a very thorough treatment of synchrotron applications and exact surface equations for a tangentially collimating diaboloid have been presented (McKinney *et al.*, 2009 ▸; Yashchuk *et al.*, 2020 ▸; Yashchuk *et al.*, 2021 ▸; Sanchez del Rio *et al.*, 2021 ▸). In this work we will investigate a sagittal collimating diaboloid. See Fig. 2 ▸ for a schematic portrayal of diaboloids.

## 2. Surface equation of a sagittal collimating diaboloid

To derive the surface equation, we follow the same procedure as used by Yashchuk *et al.* (2021 ▸). We look for a solution where the path length from the source to the tangential line focus is the same, and the outgoing rays are all parallel to the plane of symmetry. That is, all reflected rays have no *x*-direction component.

**Figure 3:**
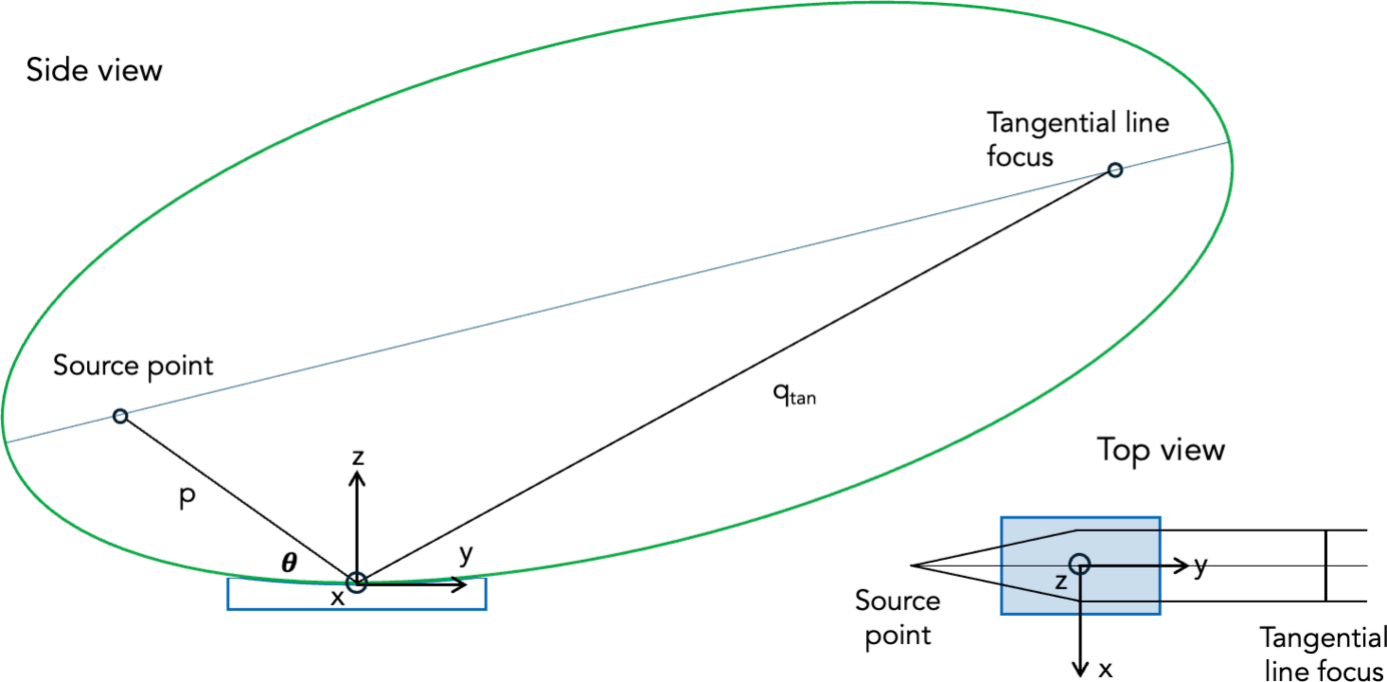
Schematic diagram of a sagittal collimating diaboloid. The origin of the coordinate system is taken at the pole of the mirror, with the *x* axis in the sagittal direction, the *y* axis in the tangential direction and the *z* axis normal to the mirror surface. In the *yz* plane of symmetry, *p* is the distance from the source point to the mirror pole, *q*_tan_ represents the distance from the mirror pole to the tangential focus and θ is the angle of incidence on the mirror at the pole.

Referring to Fig. 3 ▸, we take that a general point on the mirror surface is given by (*x*, *y*, *z*). The source point is given by (*x*_s_, *y*_s_, *z*_s_) = 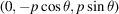. The distance *d*_1_ from the source to a general point on the mirror surface is then given by 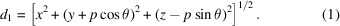
Now consider the distance of the reflected ray from the mirror surface to the tangential line focus. By inspection, it can be seen that, in order for this ray to be collimated in the sagittal direction, it must have the same *x* coordinate at the line focus that it did on the mirror surface. Therefore, the point where the reflected ray intersects the line focus is given by 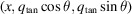. The distance *d*_2_ from the mirror surface to the line focus is then given by
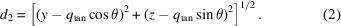
According to Fermat’s principle (Hecht, 1998 ▸), for any point on the mirror surface, the distance from the source to any given point on the tangential line focus must be the same. In the *yz* plane, it is clear that this distance is *p* + *q*_tan_. We postulate that this is true not just in the *yz* plane but for any point along the tangential line focus. From this point it is straightforward to write down the distance equation, 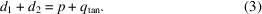
A tedious but straightforward algebraic expansion of equation (3) and collection of terms in like powers of *z* leads to a quadratic equation in the standard form of *Az*^2^ + *Bz* + *C* = 0 with 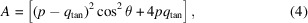

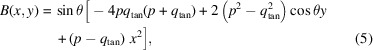

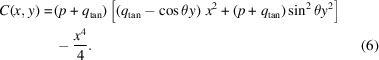
The desired real solution, which gives the surface height as a function of *x*, *y*, is given by 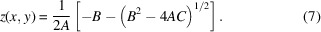
By inspection, it is also clear that, in the symmetry plane (*x* = 0), the surface shape must be an ellipse. In contrast, the surface shape of a tangentially collimating diaboloid in the symmetry plane is a parabola (Yashchuk *et al.*, 2021 ▸). These equations have been recently published without derivation by Huang *et al.* (2026 ▸).

## 3. Application of a sagittal collimating diaboloid to a soft X-ray spectrometer

**Figure 4:**
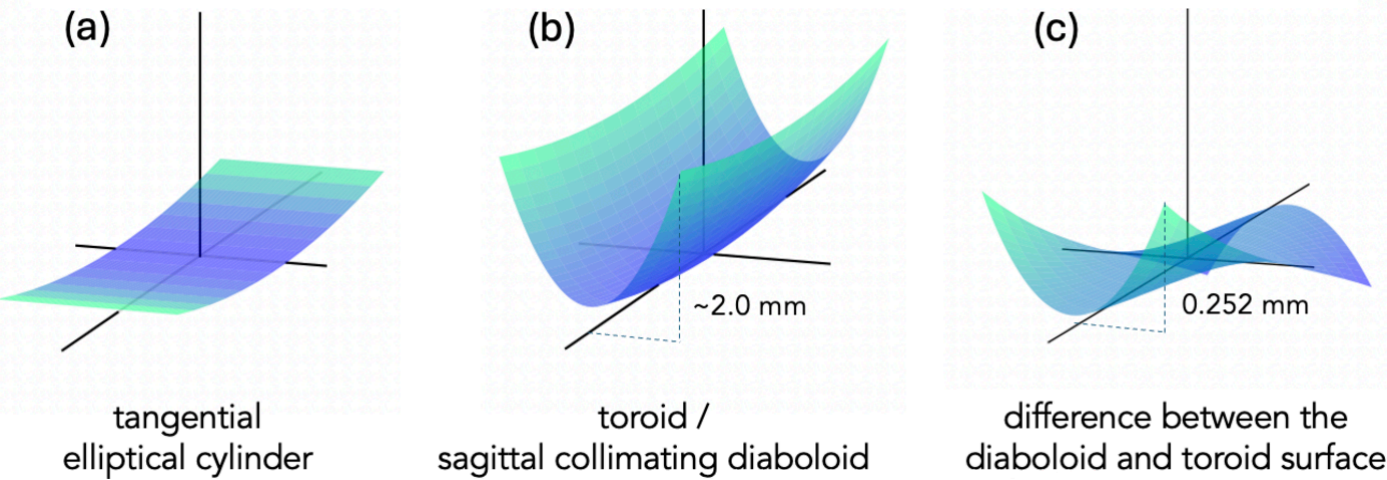
Surfaces of the three vertical focusing mirrors. (*a*) Tangential elliptical cylinder. (*b*) Toroid and sagittal collimating diaboloid. Both of these figures are drawn to the same scale. Note that, at this scale, the toroid and diaboloid surface appear nearly identical. (*c*) To illustrate the contrast between the diaboloid and the toroid, the difference between the two surfaces is plotted and the *z* scale is stretched for emphasis. The maximum difference between the two surfaces is 0.252 mm.

Now that the equation of the diaboloid shape is known, it can be applied in an example spectrometer design. As discussed in the *Introduction*[Sec sec1], we will apply the diaboloid to the Hettrick–Underwood design. We will look at three situations. Case 1 is where the vertical focusing mirror is a tangential elliptical cylinder, such that the detector width determines the horizontal acceptance of the spectrometer. Case 2 is where the vertical focusing mirror is a toroid, in an attempt to increase the horizontal acceptance by collimating the beam in the sagittal direction. Case 3 is where the vertical focusing mirror is a sagittal collimating diaboloid. The three cases are shown in Fig. 4 ▸. Note that, to the eye, the toroid and diaboloid surfaces would appear nearly identical. The contrast in the two surfaces is best illustrated by taking the difference in the surface height, which is shown in Fig. 4 ▸(*c*). The maximum height difference is only 0.252 mm.

For a concrete example we will assume some reasonable parameters. There are some technical limits on mirror manufacturability to be considered. A toroid and a sagittal collimating diaboloid require sagittal curvature which is much higher than the tangential curvature, representing a significant challenge to metrology and therefore fabrication. This is a well known consequence of Coddington’s equations (Kinslake, 1994 ▸). Discussions with state-of-the-art optics manufacturers indicate that this limit is currently ∼50 mm radius for an acceptable figure error. This limit on the sagittal radius means that the shortest spectrometer this can be applied to is about 3 m long with the vertical focusing mirror 550 mm from the source. This is short enough that, in principle, it can fit on many spectroscopy endstations if being designed from the start, although at this length retrofitting may pose problems. We will assume a source size of 5 µm × 5 µm, since modern spectroscopy endstations can achieve this order of focusing. We will aim for a moderate resolving power of 3000 at the O *K* edge (540 eV), a resolution that is suitable for a wide range of chemical and material investigations (Weinhardt *et al.*, 2013 ▸; Yin *et al.*, 2015 ▸; Qiao *et al.*, 2017 ▸; Glans *et al.*, 2017 ▸). This can be achieved by a 1300 lines mm^−1^ grating operating in outside order with α = 87.5° at 540 eV. For diffraction efficiency calculations we will take a grating blazed for maximum efficiency at 540 eV with an ideal blazed profile. The full list of design parameters is given in Table 1 ▸.

Additionally, the vertical focusing mirror will operate at a grazing-incidence angle of 3°. This angle of incidence optimizes the acceptance × reflectivity product of an Au coated mirror up to 1000 eV. Furthermore, we will take as the detector a representative soft X-ray CCD detector with a spatial resolution of 24 µm FWHM and 27.6 mm × 27.6 mm dimensions (Ghiringhelli *et al.*, 2006 ▸). For this example we will initially assume optics with no figure error.

The expressions used to determine the VLS coefficients are obtained from the literature. For the toroid VFM case, the expressions can be found in the reports by Amemiya *et al.* (1996 ▸) and Amemiya & Ohta (2004 ▸). For the elliptical cylinder and sagittal collimating diaboloid VFM cases, the virtual source has no aberration, and therefore the optical system can be viewed as a plane grating with an ideal virtual source. The expressions are found in the work of Harada *et al.* (1999 ▸), which gives the equations for a spherical grating; the case of a plane grating is derived by setting the radius of curvature to infinity.

For ray-tracing analysis we use the open source software *xrt* (Klementiev & Chernikov, 2014 ▸). The software is written in Python, which makes it easy to implement custom optical elements. The elliptical cylinder and toroid optical elements are part of the base package. We have extended *xrt* with a custom sagittal collimating diaboloid optical element.

In this context, it is not possible to design an optimized spectrometer. This can only be done when the scientific goals of energy range, resolution and efficiency are defined, and additional parameters such as source size and overall length are determined. We present this as a reasonable example to illustrate the application of a sagittal collimating diaboloid.

**Figure 5:**
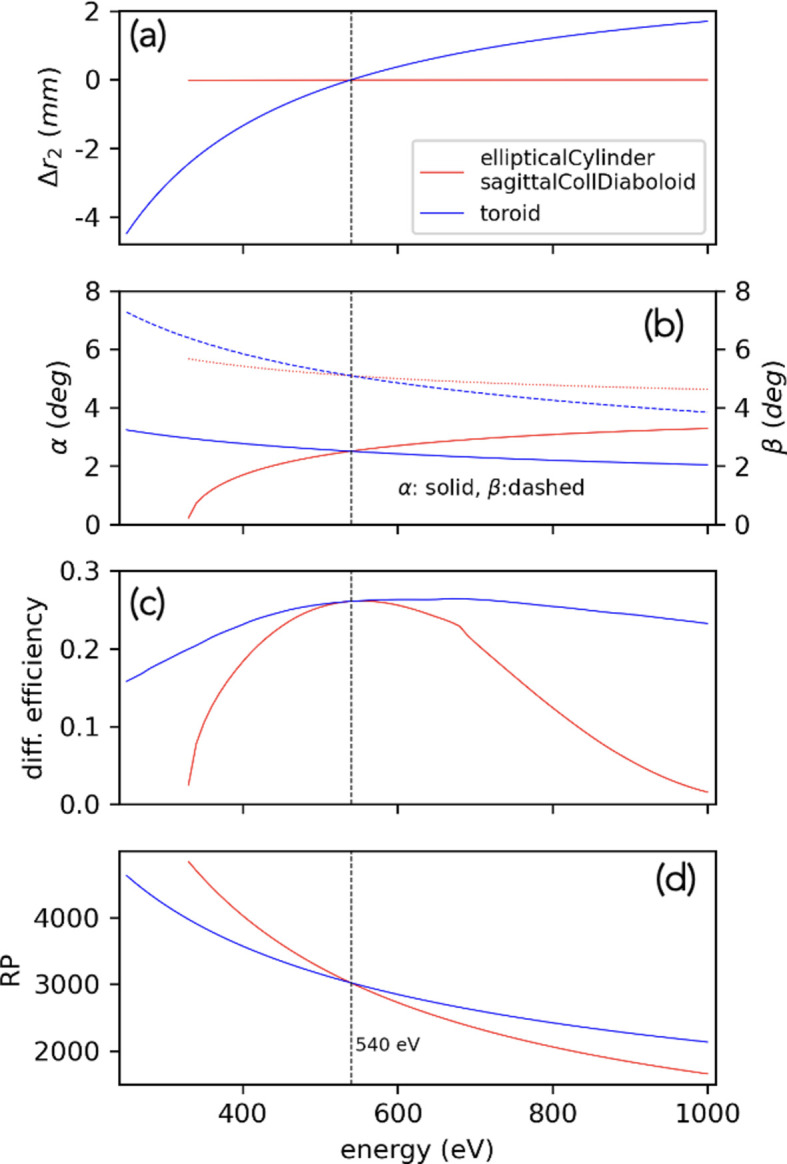
Tuning behavior of the three spectrometer designs. (*a*) The change in detector distance *r*_2_ as a function of energy required to zero aberrations up to the coma term. Since the elliptical cylinder and sagittal collimating VFM form a nearly perfect virtual image, the focal distance is essentially constant with energy. For the toroid VFM, the change in distance is still quite minimal, requiring only a few millimetres change to stay in focus. (*b*) The changes to α and β required to zero the aberrations up to the coma term. Here we plot α and β as grazing angles. Note that the behavior between the elliptical cylinder/sagittal collimating and toroid VFM are quite different. (*c*) The first-order diffraction efficiency for the blazed 1300 mm^−1^ grating. Due to the different angular tuning behavior of the elliptical cylinder/sagittal collimating and toroid VFM design, the diffraction efficiency is different. (*d*) The nominal resolving power, which again varies between the cases due to the different angular tuning of the grating.

For most soft X-ray spectrometers, a single grating is used to cover a relatively wide energy range of several hundred electronvolts or more. Therefore, the tuning properties, *i.e.* the adjustments to the grating angle and detector position to keep the image in focus, are important. These are shown in Fig. 5 ▸, and are different between the elliptical cylinder/sagittal collimating and toroidal VFM cases. The differences can be understood by considering the differences in the virtual source produced by the different mirrors. In the case of an elliptical cylinder/sagittal collimating mirror, the virtual image is aberration free for an ideal point source. In the case of a toroid, the magnification is 4.45×. Toroids are known to focus well only near unity magnification. The virtual image formed by the toroid will have significant aberrations. These are removed up to the coma term by the VLS law of the grating, but the change in distance required to keep the detector in focus [Fig. 5 ▸(*a*)] and the changes in grating angles needed to zero aberrations up to the coma term [Fig. 5 ▸(*b*)] are different. This change in the angular tuning results in a different energy dependence of the grating efficiency [Fig. 5 ▸(*c*)] and energy dependence of the energy resolution [Fig. 5 ▸(*d*)]. The resolution is the nominal energy resolution, in other words the energy resolution at which the vertical and horizontal acceptances are small enough so that aberrations introduced by the figure of the VFM are negligible. Due to the difference in angular tuning, the elliptical cylinder/sagittal collimating case will have higher nominal resolving power below 540 eV, and lower above 540 eV, relative to the toroid case.

There are some additional things to note. First, for the elliptical cylinder/sagittal collimating case, the coma aberration can be canceled only down to 330 eV, while for the toroid case the coma can be canceled down to at least 250 eV, which leads to a wider energy window in which a single grating can be used. Second, for the elliptical cylinder/sagittal collimating case, the change in detector distance from the grating is nearly zero, and even for the toroid case the change is very modest, only a few millimetres. This is one of the valuable properties of the Hettrick–Underwood design.

### 3.1. Vertical acceptance

In the vertical direction, aberrations are corrected by the VLS law on the grating. By tuning the spectrometer properly, they are zeroed up to the coma term at all energies, and up to the spherical term at 540 eV. As a result of this good, but incomplete, aberration correction, as higher-order aberration terms are not canceled, the spectrometer has a limit on the vertical acceptance, at which point the resolution starts to degrade. To determine the vertical acceptance, we ray trace each case as a function of vertical acceptance at a narrow horizontal acceptance of 0.5 mrad, which is sufficient that any resolution degradation due to horizontal aberrations is negligible.

**Figure 6:**
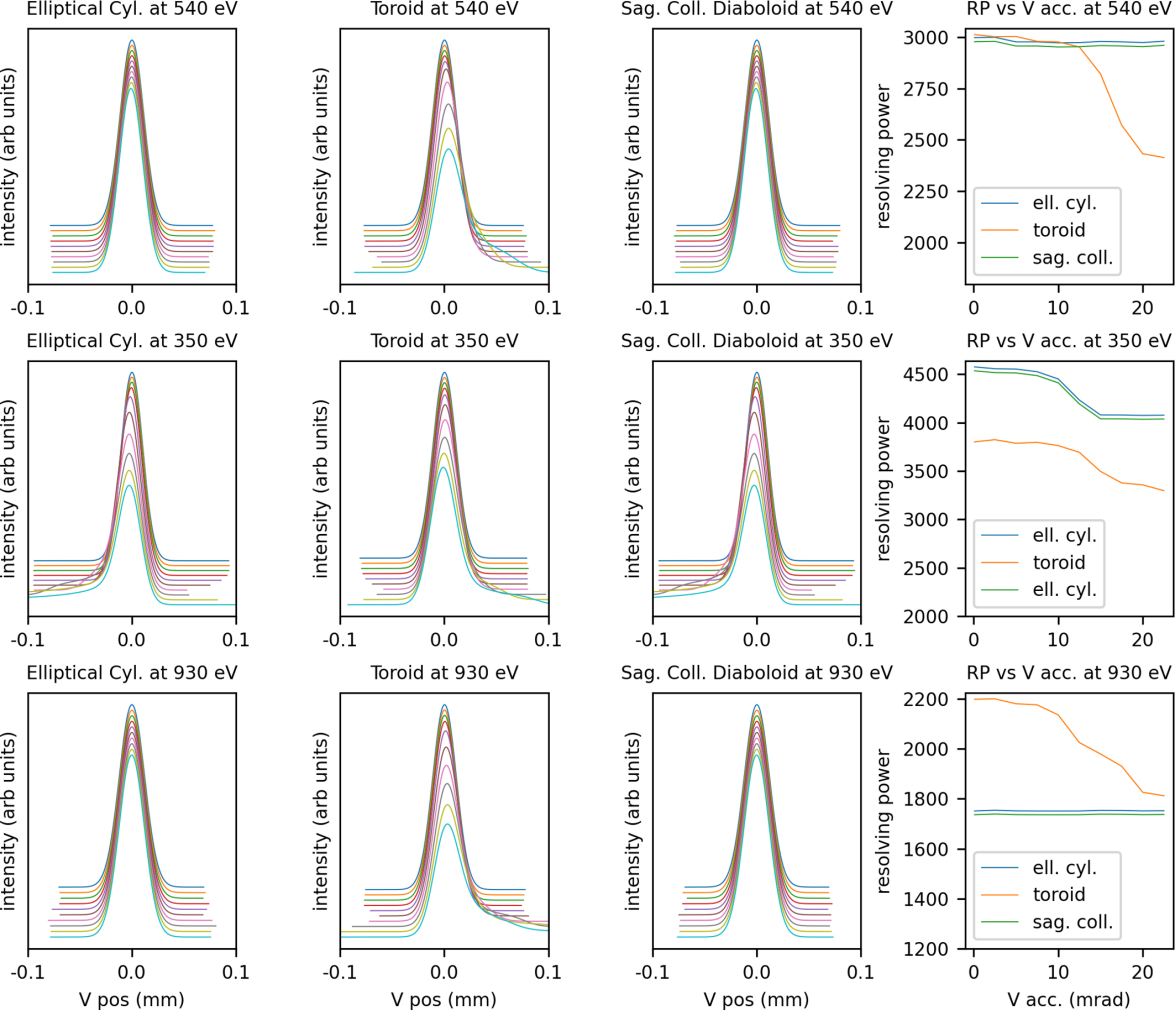
Vertical acceptance of the three spectrometer designs, (left-hand column) the elliptical cylinder, (second column) the toroid and (third column) the sagittal collimating diaboloid. Each row corresponds to a particular energy, (top) 540 eV, (middle) 350 eV and (bottom) 930 eV. The three plots starting from the left-hand side on each row show the evolution of the vertical image profile as the vertical acceptance is increased up to 22.5 mrad (curves offset for clarity). The right-hand plots show the resolving power determined by the vertical image profile as a function of the vertical acceptance. The ray-tracing images are convolved with the detector spatial resolution. The horizontal acceptance is set to 0.5 mrad, narrow enough that horizontal aberrations are negligible and do not affect the energy resolution.

Fig. 6 ▸ shows the results for the three cases at three different energies: at the design optimization energy of 540 eV, at the low end of the energy range, 350 eV, and at the high end of the energy range, 930 eV. We look first at the top row of the figure. The first three plots show the evolution of the vertical image profile as the vertical acceptance is increased up to 22.5 mrad at 540 eV. The elliptical cylinder and sagittal collimating cases behave ideally up to at least 22.5 mrad, showing no degradation in the focus. These results are summarized in the right-hand plot, which shows the resolving power as a function of the vertical acceptance. For the toroid case, however, the focus begins to degrade for acceptances above ∼10 mrad, with the resolving power decreasing by 10% (by ∼15 mrad). At the lower end of the energy range, 350 eV, we see similar behavior between the three cases. The ability of the VLS grating to cancel aberrations decreases below the optimization energy, and all designs can accept ∼10 mrad before the vertical image begins to degrade. Above 540 eV, however, the elliptical cylinder/sagittal collimating case has superior performance (bottom row in Fig. 6 ▸). These two cases can accept up to at least 22.5 mrad with no image degradation, whereas the toroid case shows image degradation above 10 mrad. Whereas for the toroid case the resolution begins to degrade above 10 mrad vertical acceptance, somewhat counterintuitively the resolving power is actually higher at 930 eV. This is because both α and β are more grazing for the toroid case at high energy, which reduces the source and detector contributions to the resolution.

### 3.2. Horizontal acceptance

**Figure 7:**
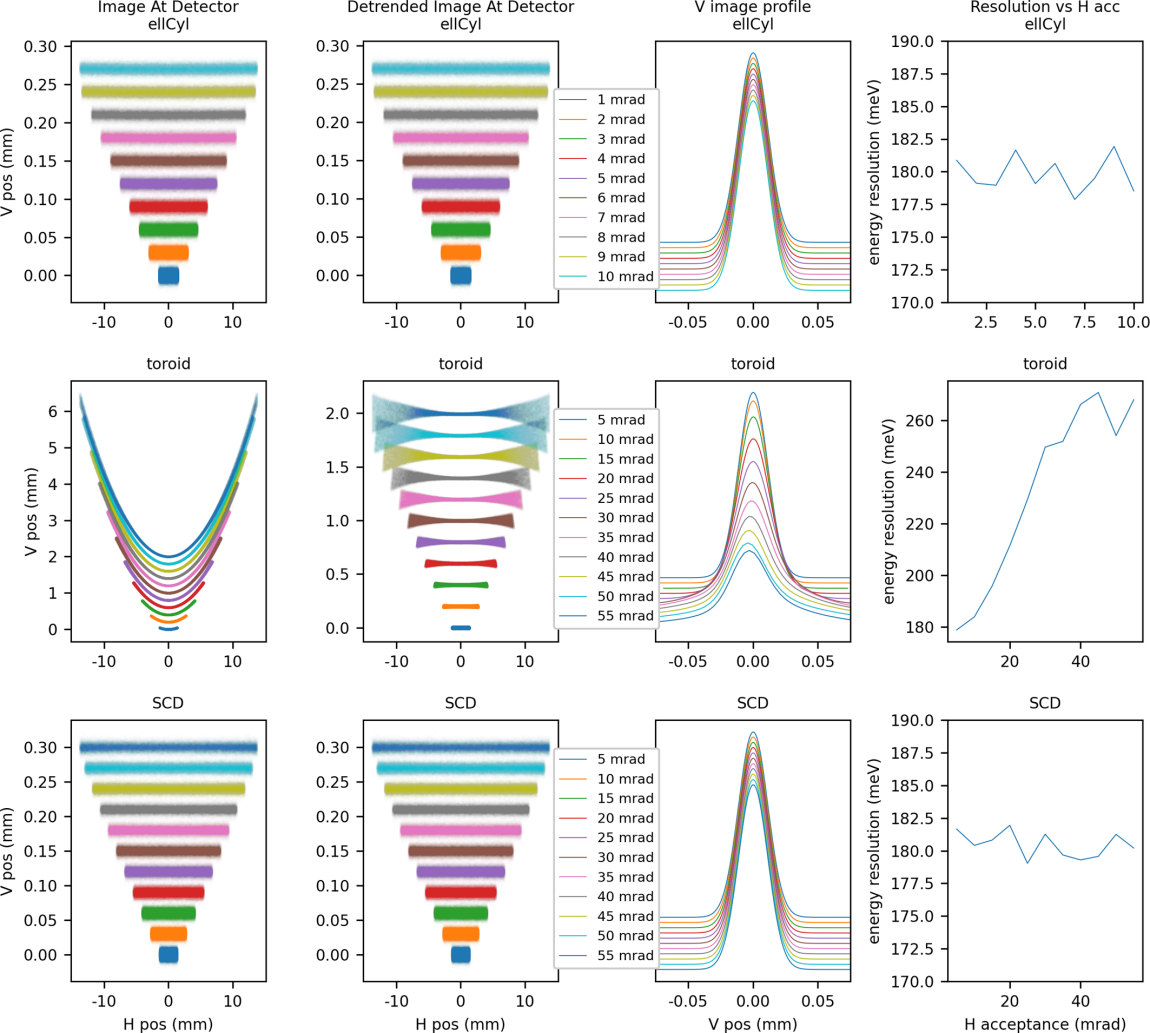
Horizontal acceptance of the three spectrometer designs at 540 eV. Each row corresponds to a particular design, (top) the elliptical cylinder, (middle) the toroid and (bottom) the sagittal collimating diaboloid. The left-hand column shows the images of the beam on the detector for an increasing series of horizontal acceptance. The second column shows the images with the curvature detrended using a second-order polynomial. The third column shows the resultant vertical beam profiles, from which the resolution is determined. The fourth column shows the resulting energy resolution as a function of the horizontal acceptance. The images are offset for clarity. The noise in the resolution plots is due to the finite number of rays used in the ray tracing.

The more dramatic difference between the three cases is the behavior as a function of the horizontal acceptance. To investigate this, we take a similar approach and ray trace each design while varying the horizontal acceptance, with the vertical acceptance fixed at 1 mrad. The results are shown in Fig. 7 ▸.

For the elliptical cylinder case (top row), the image at the detector is very slightly curved, although the finite source size obscures the curvature. The image maintains its quality up to 9.2 mrad horizontal acceptance, at which point the acceptance is limited by the detector width. The resolution remains constant up to this point.

For the toroid case (center row), the image quality is strongly affected by the horizontal acceptance. In this case, there is a large curvature to the image. This in itself is not a problem, because the curvature can be removed by detrending with a second-order polynomial. The detrended images are also shown. It is readily seen that the resolution degrades as the horizontal acceptance increases. In this case, the spectrometer can accept ∼17 mrad before the energy resolution degrades by 5%. The spectrometer can accept up to 61 mrad geometrically in the horizontal direction, at which point the detector limits the acceptance but the resolution suffers.

In the case of the sagittal collimating diaboloid (bottom row), the image formed is strictly a straight line, as the beam is well collimated in the sagittal direction. The spectrometer can accept up to 61 mrad in the horizontal direction, at which point the detector limits the acceptance. There is no degradation in the energy resolution up to this point. This illustrates the effectiveness of the diaboloid optic in increasing the spectrometer acceptance.

### 3.3. Detailed numerical comparison

**Figure 8:**
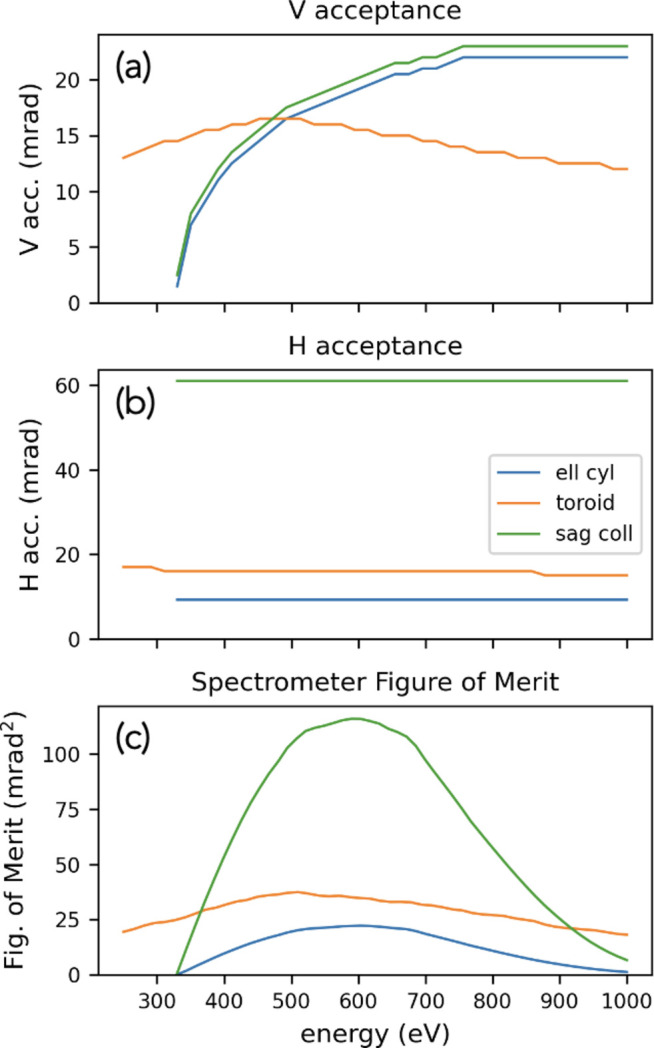
Numerical comparisons of the throughput of the three spectrometer designs, with the elliptical cylinder in blue, the toroid in orange and the sagittal collimating diaboloid in green. In the elliptical cylinder and sagittal collimating cases, the (*a*) V and (*b*) H acceptances are determined by the acceptance of the optics, while in the toroid case they are limited by the degradation to the energy resolution over the full energy range. The resulting figure of merit is shown in panel (*c*). Note that the figure of merit is in quantitative units of mrad^2^.

To compare the several spectrometer designs quantitatively, each design was ray-traced to determine the exact geometric acceptance over the full energy range. The results are shown in Fig. 8 ▸. In the elliptical cylinder/sagittal collimating cases, the horizontal and vertical acceptances are determined only by the energy-dependent acceptance of the optics. In the toroid case, the energy resolution starts to degrade before the optics are filled; we set the limit to the acceptance at the point where the resolution degrades by more than 10% of the design resolution.

Considering the vertical acceptance, the elliptical cylinder/sagittal collimating cases perform worse at lower energies where the incident angle is more grazing on the grating, but performs better at higher energies where the angle on the grating is less grazing. The toroid vertical acceptance is limited to ∼15 mrad over the full energy range by aberrations.

Considering the horizontal acceptance, the elliptical cylinder case is limited to 9.2 mrad by the detector width. The toroid case is limited to ∼17 mrad by aberrations. In the sagittal collimating case, the horizontal acceptance is again limited by the detector width, but due to the horizontal collection which results from the sagittal curvature, 61 mrad can be collected in the horizontal without aberrations in the image.

Fig. 8 ▸(*c*) shows the figure of merit, which is defined as the geometric acceptance × VFM reflectivity × diffraction efficiency. Note that this results in absolute units of mrad^2^, and thus can be compared quantitatively with any other spectrometer design. Over the majority of the energy range, the sagittal collimating diaboloid design is superior. At the peak in efficiency, it is 5.6 times more efficient than the elliptical cylinder design and 3.4 times more efficient than the toroid design, with no sacrifice to the energy resolution.

### 3.4. Practical considerations regarding sagittal collimating diaboloids

#### 3.4.1. Finite source size aberrations

**Figure 9:**
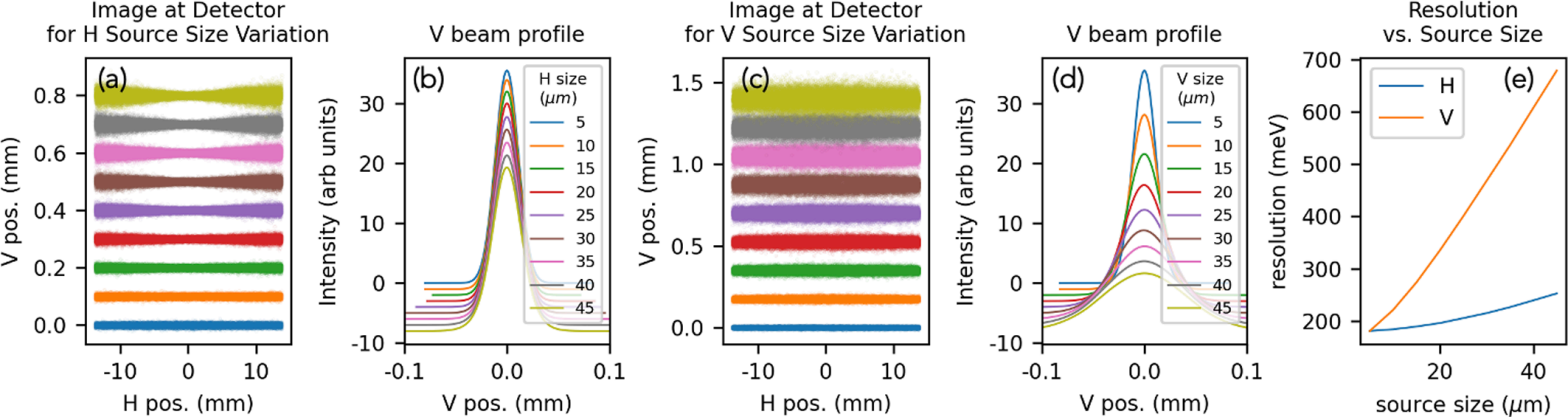
Effects of finite H and V source size at 540 eV for the sagittal collimating design. (*a*) The image at the detector for increasing H source size. (*b*) The resulting V profile of the beam. (*c*) The image at the detector for increasing V source size. (*d*) The resulting V profile of the beam. The curves are offset for clarity. (*e*) The resulting energy resolution as a function of the H and V source sizes. The H and V acceptances are set to the full value accepted by the spectrometer.

We have defined a diaboloid as an optic that is aberration free for point and line sources. That does not mean that there are no aberrations to consider. Sources are not ideal points or lines; they have a finite size. A frequently occurring refocusing situation for beamline endstations is the use of a Kirpatrick–Baez (KB) mirror pair, which for geometric reasons necessarily has different demagnifications in the horizontal and vertical directions, which often results in different horizontal and vertical focus sizes. The question arises as to what happens if the horizontal source size is larger than the vertical. For the example spectrometer, we have assumed a symmetric source size of 5 µm × 5 µm, which is small enough that the field-dependent aberrations are negligible. In Fig. 9 ▸, the focusing behavior of the spectrometer is investigated as a function of the horizontal and vertical source sizes. For these calculations, the energy is 540 eV and the H and V acceptances are set to the full acceptance of the spectrometer.

Fig. 9 ▸(*a*) shows the image at the detector as the H source size is increased. At 5 µm source size, the aberrations are negligible. However, as the source size is increased the image begins to develop a ‘bow tie’ appearance. Due to the sagittal curvature, the design is sensitive to the H source size. Fig. 9 ▸(*b*) shows the resulting V beam profile, and the resulting energy resolution versus H acceptance is shown in Fig. 9 ▸(*e*). If we set 10% of the optimal as a tolerable degradation to the energy resolution, the spectrometer can tolerate a source size up to ∼23 µm FWHM in the horizontal. In this respect, this design is more sensitive to a design with a separate H collection mirror which, due to the nearly complete separation of H collection and V dispersion, is very insensitive to the H source size. However, with the many current and planned synchrotron upgrades [see *e.g.* Karantzoulis *et al.* (2018 ▸), Chenevier & Joly (2018 ▸), White *et al.* (2019 ▸), Kerby (2023 ▸), Conroy (2024 ▸), Tanaka *et al.* (2024 ▸) and Willmott & Braun (2024 ▸)] with highly reduced H emittances and resulting smaller H focus sizes at the endstation, this should not be a significant drawback.

Fig. 9 ▸(*c*) shows the image at the detector as the V source size is increased. The detector image increases as well. Note that this is not due to aberrations; it is due to the inherent vertical imaging properties of the spectrometer. As the vertical source size is increased, the image size naturally increases, with a corresponding decrease in the energy resolution. Fig. 9 ▸(*d*) shows the vertical profile of the image at the detector, and Fig. 9 ▸(*e*) summarizes the energy resolution versus the V source size. The energy resolution changes linearly with source size above ∼15 µm, indicating that in this regime the spectrometer resolution is source size limited, and that the V imaging remains good with minimal aberrations.

#### 3.4.2. Figure error

The manufacture of arbitrary surface figures is in principle a solved problem; the advent of deterministic polishing techniques such as ion beam figuring with feedback from metrology allows the manufacture of any desired figure (Yashchuk *et al.*, 2020 ▸; Cocco *et al.*, 2022 ▸; Wang *et al.*, 2023 ▸). The limiting factor is not the polishing but the metrology. High sagittal curvatures remain a known challenge to producing aspherical figures (Gevorkyan *et al.*, 2017 ▸; Munechika *et al.*, 2022 ▸).

The sagittal collimating diaboloid used in the current design has a sagittal radius at the mirror pole of 57.6 mm, and we have purposely made the spectrometer large enough to keep the sagittal radius larger than 50 mm. For a diaboloid with similar sagittal curvature, ∼3 and ∼0.5 µrad sagittal and tangential slope errors are achievable. In this design, the figure error would result in a broadening of the vertical image by ∼7 µm, which, combined in quadrature with the detector spatial resolution of 24 µm, would result in less than 5% degradation in the energy resolution, which could easily be compensated for with a comparable increase in the line density of the grating. However, the impact of the figure error on the resolution will be design specific and will ultimately result in limits to the resolution that can be achieved.

### 3.5. Alignment

**Figure 10:**
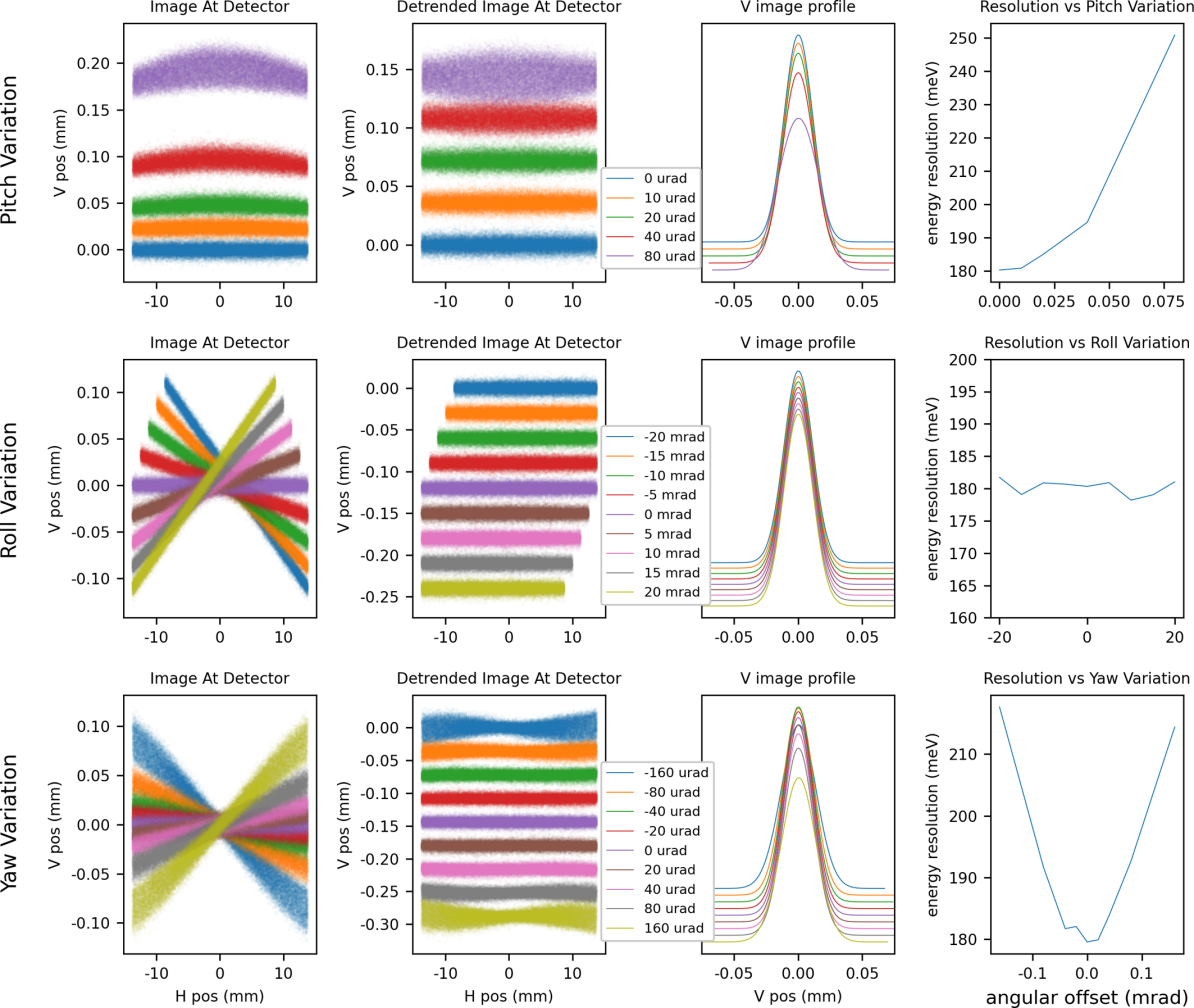
Sensitivity of the sagittal collimating design to angular misalignments. The top row shows the effect of pitch misalignment. The left-hand panel shows the image at the detector for increasing misalignment. The second panel shows the image detrended with a second-order polynomial, which can remove the curvature. The third panel shows the vertical beam profile and the right-hand panel shows the resulting energy resolution as a function of the angular misalignment. The second row shows the same for roll misalignment and the third row the same for yaw misalignment. Images are offset for clarity. The noise in the resolution plots is due to the finite number of rays used in the ray tracing.

The presence of sagittal curvature on the diaboloid prompts the question as to its sensitivity to angular misalignment. The sensitivity is investigated by ray tracing and the results are shown in Fig. 10 ▸. The top row of the figure shows the effects of pitch misalignment. In the left-hand panel, the image at the detector is shown for increasing angular misalignment. Mis­alignment causes both a curvature and a distortion to develop. The curvature can be detrended with a second-order polynomial, which is shown in the second panel, but the distortion is not removed. The vertical profile of the image is shown in the third panel, and the resulting energy resolution as a function of angular misalignment is shown in the right-hand panel. If we take 5% resolution degradation as tolerable, then the pitch must be aligned to within ±30 µrad. As modern synchrotron mirror pitch mechanisms can routinely reach sub-50 nm resolution and stability, this level of alignment is achievable.

The second row in Fig. 10 ▸ shows the corresponding results for misalignment of the VFM roll. This results in a tilting of the image on the detector as shown in the left-hand panel, which can be completely removed by detrending with a linear fit. No distortion to the vertical profile is seen, and the resolution remains unaffected up to at least ±20 mrad of roll misalignment. The diaboloid is thus very forgiving for mis­alignments in roll.

The third row in Fig. 10 ▸ shows the corresponding results for yaw misalignment. Due to the sagittal curvature, it is anticipated that the resolution will be sensitive to yaw, and this is indeed the case. Misalignment in yaw leads to both a tilt and a distortion to the image at the detector. The tilt can be removed by detrending but the distortion cannot. The resulting energy resolution versus angular misalignment is shown in the right-hand panel. Again, if a 5% degradation in energy resolution is deemed tolerable, the yaw must be aligned to within ±70 µrad. This is significantly more sensitive than a spectrometer with an elliptical cylinder VFM, which, due to the lack of sagittal curvature, has milliradian tolerances on the yaw. An accurate mechanism for yaw alignment must be included in the spectrometer design.

## 4. Summary

Photon emissions through either fluorescence or RIXS processes are rare events in the soft X-ray regime, necessitating continuous improvement in spectrometer design. To that end, we have introduced a new optical figure, a sagittal collimating diaboloid. We foresee that this optic will be very useful for improved Hettrick–Underwood spectrometers. A single optic can be used both to collect photons in the horizontal direction and focus them in the vertical direction in an aberration-free manner. This leads to a significant improvement in collection efficiency relative to a design with no horizontal collection optics or with a toroid as the vertical focusing mirror.

Current capabilities in the manufacture of such an optic allow its implementation in a 3 m long or larger spectrometer, while further advances in metrology are anticipated to enable even more compact spectrometers using the diaboloid optic.

## Figures and Tables

**Table 1:** Parameters for an example spectrometer

Common parameters
Source size = 5 µm × 5 µm FWHM
*r*_*a*_ = 550 mm
*r*_*b*_ = 100 mm
*r*_2_ nominal = 2350 mm

Vertical focusing mirror
200 mm long by 27.6 mm wide optical dimensions
Grazing incidence angle = 3°
Au coating
Elliptical cylinder: *p* = 550 mm, *q*_tan_ = 2450 mm
Toroid: ρ = 57.5696 mm, *R* = 17164.7 mm
Sagittal collimating diaboloid: *p* = 550 mm, *q*_tan_ = 2450 mm

Grating
Outside diffraction order, α = 87.5° at 540 eV
200 mm long by 27 mm wide optical dimensions
Au coating
Ideal blazed profile, blaze angle = 1.3°, central line density = 1300 mm^−1^
VLS law for Case 1 and Case 3: *a*_1_ = 1.10368 mm^−2^, *a*_2_ = 7.0103 × 10^−4^ mm^−3^, *a*_3_ = 3.9483 × 10^−7^ mm^−4^
VLS law for Case 2: *a*_1_ = 1.10368 mm^−2^, *a*_2_ = −1.1391 × 10^−3^ mm^−3^, *a*_3_ = −5.298 × 10^−7^ mm^−4^

## Data Availability

All available data are published within this article.

## Footnotes

1 For simplicity, we will refer to the dispersion direction as vertical and the non-dispersion direction as horizontal.
